# Mode manipulation and near-THz absorptions in binary grating-graphene layer structures

**DOI:** 10.1186/1556-276X-9-90

**Published:** 2014-02-21

**Authors:** Haiming Yuan, Hanning Yang, Pengzi Liu, Xiangqian Jiang, Xiudong Sun

**Affiliations:** 1 Department of Physics, Harbin Institute of Technology, Harbin 150001, China; 2 Key Lab of Micro-Optics and Photonic Technology of Heilongjiang Province, Harbin 150001, China

**Keywords:** Graphene surface plasmons, Excitation condition, Multilayer structure absorption

## Abstract

The excitation and absorption properties of grating coupled graphene surface plasmons were studied. It was found that whether a mode can be excited is mainly determined by the frequency of incident light and the duty ratio of gratings. In the structure consisting graphene bilayer, a blueshift of the excitation frequency existed when the distance between neighbor graphene layer were decreased gradually. In graphene-grating multilayer structures, a strong absorption (approximately 90% at maximum) was found in near-THz range.

## Background

Recently, a lot of work has been done based on graphene due to its unique properties in electric, magnetic, thermal, etc. [1-3]. Graphene is carbon atoms arranged in a two-dimensional honeycomb lattice, in which the electrons behave like massless Dirac fermions with linear dispersion [4,5]. Graphene has strong plasmonic effects which can be modified by gating, by doping, and so on [2]. A controllable optical absorption was also found in structured graphene [6,7].

Up to date, the graphene is modeled usually to be an extremely thin film with a conductivity *σ*, which consists of both intraband and interband from Kubo formula [7-9]. The intraband conductivity with Drude type plays a leading role when ℏ*ω*/*μ*_c_ was small [10]. Both transverse electric (TE) and transverse magnetic (TM) have the dispersion relations at monolayer graphene with dielectric materials on two sides [10-12]. In other words, the charge carriers coupling to electromagnetic waves will produce a new surface wave, namely graphene surface plasmons (GSPs).

In the previous works, many numerical approaches were used to study the structured graphene, for example the finite element method (FEM) [13], finite difference time domain (FDTD) [14], and others [6,15]. A strong plasmonic response of graphene has been demonstrated in a square-wave grating with a flat graphene on top [15]. In which, the graphene-based plasmon response lead to a 45% optical absorption. In a periodic array of graphene ribbons, remarkably large GSPs result in prominent optical absorption peaks [13]. In multilayer graphene, the absorption spectrum can be decomposed into subcomponents [6], which is helpful in understanding the behavior of GSP coupling.

In this paper, we studied the binary grating bounded by graphene on both sides. The rigorous coupled wave analysis (RCWA) [16,17] was used the first time as we know to characterize the graphene-containing periodic structures. The excitation condition and excitation intensity seemed to be influenced by the grating constant, duty ratio and the distance between the graphene layers. When introducing more graphene layers into the structure periodically, a strong absorption band was found in the near-THz range.

## Methods

### Electromagnetic mode of binary grating-graphene

Previous research has shown that the conductivity of graphene came from the contribution of intraband and interband [18-22]. The interband conductivity tends to be ignorable when ℏ*ω* ≾ *μ*_c_ (see [10]). Then the intraband conductivity can be expressed as [23]

(1) $$\sigma \left(\mu_{c}\right)=i\frac{e^{2}k_{B}T}{\pi \hbar^{2}\left(\omega +i\tau^{-1}\right)}\left[\frac{\mu_{c}}{k_{B}T}+2\ln\left(e^{-\mu /k_{B}T}+1\right)\right]$$

where *μ*_c_ is the chemical potential, relating to the electron density. Equation 1 became a Drude type when *μ*_c_/*k*_B_*T* ≫ 1, i.e.

(2) $$\sigma \left(\mu_{c}\right)=\frac{e^{2}\mu_{c}}{\pi \hbar^{2}\left(\omega +i\tau^{-1}\right)}i$$

First, we studied graphene layer positioned at the interface of two half planes with permittivity *ε*_1_ and *ε*_2_, respectively. The electric induced current on graphene layer resulted in a magnetic field difference, which led to the coupled GSP on graphene layer. Using Maxwell equation and boundary condition, GSP modes were proved to existed for both TE and TM polarization [12,23-25]. For TE mode, the dispersion relation was as follows:

(3) $$\sqrt{\beta^{2}-k_{0}^{2}\varepsilon_{2}}+\sqrt{\beta^{2}-k_{0}^{2}\varepsilon_{1}}-i\omega \mu_{0}\sigma =0,$$

and for TM mode it became

(4) $$\frac{\varepsilon_{1r}}{\sqrt{\beta^{2}-k_{0}^{2}\varepsilon_{1}}}+\frac{\varepsilon_{2r}}{\sqrt{\beta^{2}-k_{0}^{2}\varepsilon_{2}}}-\frac{\sigma }{i\omega \varepsilon_{0}}=0.$$

Because the imaginary part of conductivity (2) was positive, no solution of Equation 3 was found in real, which meant the TE mode GSP could not be excited.

For TM mode, put Equation 2 into Equation 4, we found

(5) $$\omega \left(n_{\text{eff}}\right)=\frac{e^{2}\mu_{c}}{\pi \hbar^{2}\varepsilon_{0}c}\left(\frac{\varepsilon_{1r}}{\sqrt{n_{\text{eff}}^{2}-\varepsilon_{1r}}}+\frac{\varepsilon_{2r}}{\sqrt{n_{\text{eff}}^{2}-\varepsilon_{2r}}}\right).$$

**Figure 1:**
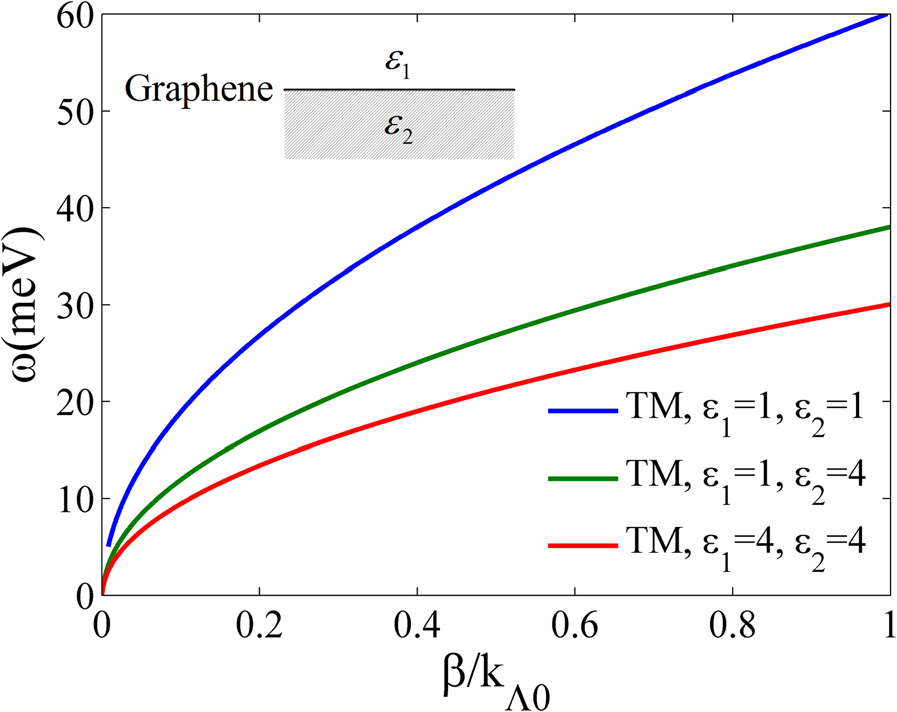
**Dispersion relations of graphene surface plasmons (GSPs) on monolayer graphene with different material on two sides.** Here, we use the graphene parameters of *μ*_c_ = 0.2 eV, *τ*^-1^ = 1 meV.

Here, we defined *n*_eff_ = *β*/*k*_0_ = *βc*/*ω* as the effective index of GSP. After making a transformation of (*ω*, *n*_eff_) → (*ω*, *β*), the dispersion relations were obtained and plotted in Figure 1. The wave vector was normalized by *k*_Λ0_ = 2*π*/*λ*_0_, *λ*_0_ = 1 μm. As a local mode, GSP modes were same as the surface plasmon polaritons (SPPs). They cannot be excited directly from the air. And in our work, gratings were used to provide an external wave vector to match the phase condition.

### Rigorous coupled wave analysis in graphene-containing structures

**Figure 2:**
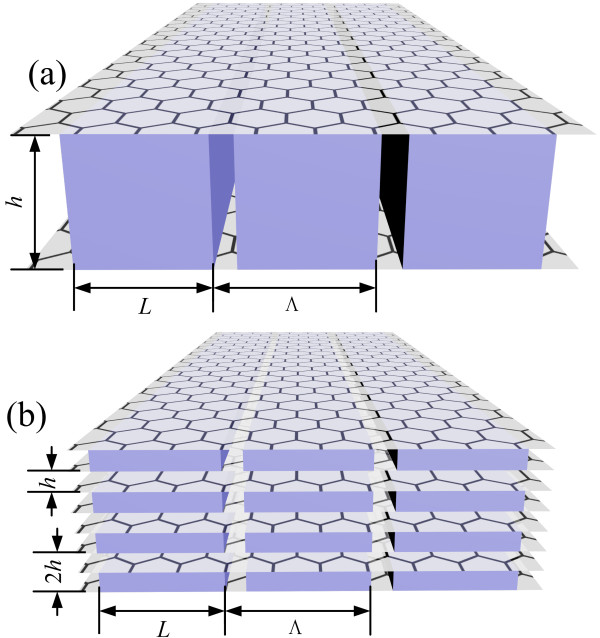
**Binary grating graphene structures. (a)** The bilayer graphene structure. **(b)** The multilayer graphene structure. *h* is the grating layer thickness. *Λ* is the period of grating. *L*_1_ is the width of dielectric with *ε*_1_. *L*(*L*_2_) is the width of dielectric with *ε*_2_. The duty ratio is *f*_2_ = *L*/Λ, and *f*_1_ = 1 - *f*_2_. In this paper, we simply set *ε*_1_ = 1 and *ε*_2_ = 4.

In Figure 2a, we used *h* to be the depth of grating (thickness of gratings). The *h* was also the distance between two graphene layers. In multilayer structures of Figure 2b, 2 *h* was the longitudinal period. The structures were designed to only contain two kinds of interfaces.

In common, the conventional RCWA based on the Floquet's theorem [26] was unable to be used for the graphene-containing structures as the electric field will induce a current with current density **J** = *σ***E**, while graphene was included.

In RCWA, the field was expanded into the form of

(6) $$E_{x}=\sum_{n}E_{x}\left(n\right)e^{i\left(\beta_{n}x-\omega t\right)},\;H_{y}=\sum_{n}H_{y}\left(n\right)e^{i\left(\beta_{n}x-\omega t\right)}.$$

So the current density **J** can also be expended to the sum of spatial harmonics with different wave vector components. To obtain the reflection, transmission, absorption, field distribution, and other optical properties of such structures as shown in Figure 2, a nonzero item must be included in the boundary condition of *H*_y_ field considering the induced current,

(7) $$H_{y+}-H_{y-}=\sigma E_{z}.$$

According to the principle of superposition, *H*_y_ will also be continuous at the interface if each spatial harmonics subcomponent satisfied the boundary conditions independently,

(8) $$H_{y}\left(n,x,y_{0+}z\right)-H_{y}\left(n,x,y_{0-}z\right)=\sigma E_{z}\left(n,x,y_{0}z\right),$$

in which *n* was the order, ± in subscripts represented approaching to *y*_0_ from two different directions. After the modification on the RCWA program, we can utilize it to deal with the graphene-containing structures.

## Results and discussion

### Phase matching condition

**Figure 3:**
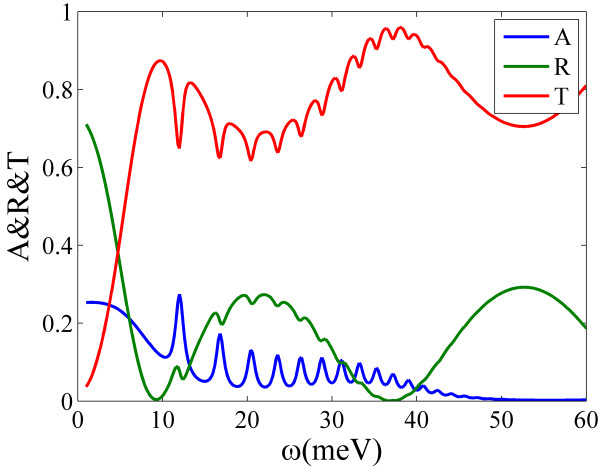
**Attenuated total reflection of the structure in Figure**2**a.** *Λ* = 11 μm, *L* = 10 μm, so the duty ratio is 10/11. Each of the absorption peaks (on blue solid line) corresponds to a GSP mode.

For a structure with a binary grating bounded by graphene layers on two sides shown in Figure 2a, the attenuated total reflection spectrum is plotted in Figure 3 using the modified RCWA method when it was illuminated normally. A set of absorption peaks each corresponding to a GSP mode was shown in blue solid line. From left to right, each peak corresponding to a GSP mode ordered with 1, 2 …

In the structure shown in Figure 2a, there exist two kinds of interfaces, i.e., *ε*_1_-graphene-*ε*_1_ and *ε*_1_-graphene-*ε*_2_. When GSP is propagating along the interfaces, the phase shifts were *φ*_1_ and *φ*_2_ for the two kinds of interfaces, respectively. *δ* was the total phase loss considering two abrupt phase changes when GSP propagates across the joints between the two kinds of interfaces in a grating period. At the excitation frequency, the phase change in a grating period should satisfy the relation

(9) $$\varphi_{1}+\varphi_{2}+\delta =2\pi N.$$

which was known to be the phase matching conditions [27,28]. In Equation 9, *N* is the integer and can be rewritten as

(10) $$N\left(\omega \right)=\frac{1}{k_{\Lambda }}\left[\beta_{1}\left(\omega \right)f_{1}+\beta_{2}\left(\omega \right)f_{2}+\frac{\delta }{\Lambda }\right],$$

where *f*_2_ = *L*/*Λ* and *f*_1_ = 1 - *f*_2_, *β*_1_ and *β*_2_ were the wave vectors of GSP on two kinds of interfaces, respectively. When *N* was a nonnegative integer, the GSP mode could be excited, and *N* can be defined as the order of surface modes. The resonant frequencies can be obtained both from absorption spectrum in Figure 3 and theoretically from Equation 10 (*δ* = 0). They were given in Table 1 and agreed well for high order modes. But for low order modes, some deviations existed between numerical and theoretical caused by the coupling of GSPs on two graphene layers.

**Table 1:** The resonant frequency of different orders

**Order of GSP (*N*)**	**1**	**2**	**3**	**4**	**5**	**6**	**7**	**…**
*ω*_0_ (meV) (RCWA)	11.9	16.7	20.5	23.7	26.3	28.9	31.1	…
*ω*_1 _(meV) (theoretical)	11.70	16.61	20.38	23.55	26.34	28.86	31.18	…

*ω*_0_ was the numerical results obtained by RCWA. *ω*_1_ was the theoretical results from Equation 10.

**Figure 4:**
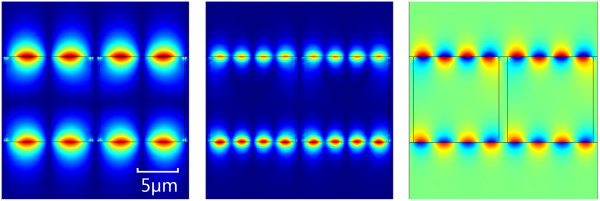
**Field distributions of |*E***_**y**_**| of first (11.9 meV, left) and second (16.7 emV, middle) order GSP modes.** The last figure was real part of *E*_y_ of second order.

The field distributions of orders 1 and 2 of the structure in Figure 2a were given in Figure 4. It was indicated that the GSP field distributions had nodes as standing wave because the GSP modes propagating in two directions were excited simultaneously.

### Duty ratio and stand wave interference

**Figure 5:**
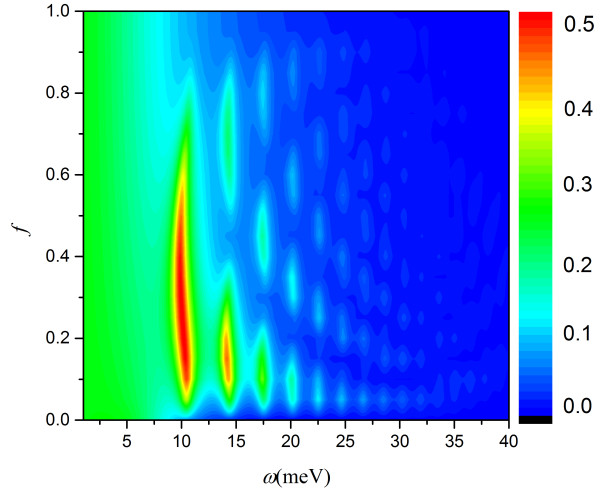
Absorption spectra for duty ratio vs the frequency fixing the light path of grating period.

By using the modified RCWA, the absorption spectrum was obtained in Figure 5 when varying *f*, where *f* = *φ*_1_/(*φ*_1_ + *φ*_2_), *φ*_1_, *φ*_2_ had the same meaning as Equation 9. From the discussion above, when the phase match conditions were satisfied, GSPs could be coupled and absorption peaks should appear. But some break points appeared as shown in Figure 5, which meant that GSPs cannot be excited at this frequency and this duty ratio and it was reasonable to believe that there must be other process related to *f* of controlling the excitation of GSP.

**Figure 6:**
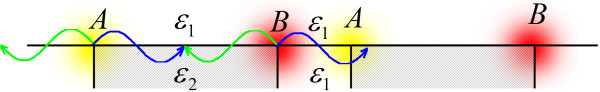
**Corners of grating will become the scatting points of the incident light which was the source of GSPs.** These scatting points can be divided into two kinds due to the geometric symmetry, which is A and B. Each scatting point will scatter into two GSP modes propagating in two directions (blue and green).

From the field distributions in Figure 4, each corner of the grating was a singular point of field and these scatting points became the sources of surface wave, as Figure 6 shown. In periodic, we only need to consider the scatting in one period, i.e., A and B. Each scatting point will couple to two GSP modes propagating in two directions. So, the field can be expressed in four terms, which is [28,29]

(11) $$\begin{array}{ll} \;F & =F_{0}e^{i\left(k_{0}x-\omega t\right)}+F_{0}e^{i\left(-k_{0}x-\omega t\right)}+F_{0}e^{i\left[k_{0}\left(x-x_{0}\right)-\omega t+\pi \right]}\\ & \;+F_{0}e^{i\left[-k_{0}\left(x-x_{0}\right)-\omega t+\pi \right]}\\ & =4F_{0}\sin\left(k_{0}x-\frac{k_{0}x_{0}}{2}\right)\sin\frac{k_{0}x_{0}}{2}e^{-i\omega t}. \end{array}$$

First two terms were GSP excited by one set of points (A in Figure 6) with two propagating directions (blue and green) and the last two terms were that from another set of points (B in Figure 6), where *x*_0_ is the distance of A and B in the form of light path (*k*_0_*x*_0_ = *L*_1_β_1_ = *φ*_1_ = (*φ*_1 +_*φ*_2_)*f* = 2*πNf*). Because in real space, different interfaces (*ε*_1_/*ε*_1_ and *ε*_1_/*ε*_2_) had different propagating constants, the expression might be complex. Here, the light path of *x* was used. It is found that scatting points A and B had a phase difference of *π*. This was caused by the different geometric symmetries. From Equation 11, when sin(*k*_0_*x*_0_/2) = 0, i.e., *f = m/N* ( *m* = 0, 1, …, *N*), field amplitude *F* would always be 0, which meant that the field cannot be excited. It was a cancelation process of two sets of standing waves that are coherent. So, for GSP mode of *N*, *N* + 1 of none absorption points appeared.

### Coupling of GSPs on different graphene layers and resonant frequency shift

**Figure 7:**
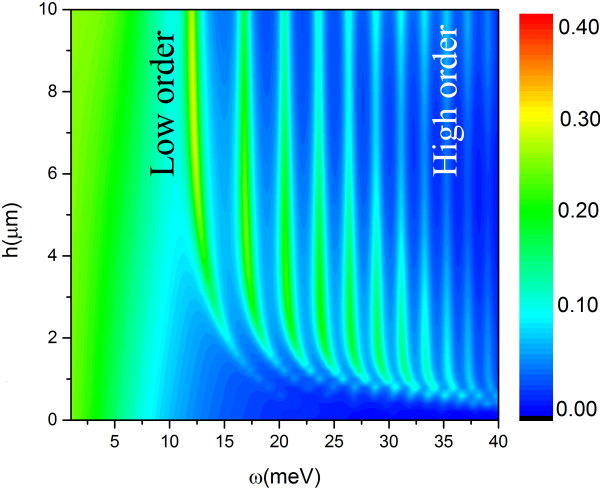
The absorption spectrum for various grating thickness.

From Table 1, we can see that for higher order modes, the consistency between the theory and the numerical results from RCWA was better than that of the lower order modes. It was because the structure consists of bilayer of graphene and there could be interaction between GSP modes on neighbor graphene layers determined by the depth of the grating. In order to understand the behavior of GSPs coupling, in Figure 7, the absorption spectra were given as a function of the grating deepness *h*. A blueshift of absorption peaks was found when the grating became thin. The oscillator model is used to describe this phenomenon of spectrum blueshift [30,31].

(12) $${\ddot{x}}_{\text{upper}}+\gamma {\dot{x}}_{\text{upper}}=F_{0}e^{-i\left(\omega t-\varphi_{n}\right)}-\omega_{0}^{2}x_{\text{upper}}-\kappa \left(n,h,\Delta \theta \right)x_{\text{lower}}$$

In Equation 12, κ(*n*, *h*, ∆θ) was the coupling coefficient and *n*, *h*, and ∆*θ* were order of GSP mode, thickness of grating, and phase difference of GSPs on two graphene layers, respectively. Essentially, the GSPs were surface waves so they interact with each other via evanescent interactions, and the coupling intensity decayed exponentially with *h* increasing. For fixed *h*, the lower order modes had larger skin depth (stronger coupling intensity) than the higher orders; then, the stronger coupling resulted in a large spectra shift. The phase difference of ∆*θ* also had affection to the absorption frequencies. However, in our case, the wavelength (15 meV ~ 82.8 μm) was much larger than the thickness of grating layer (*h* = 10 μm), it is reasonable to assume ∆*θ* is approximately 0. This can also be obtained clearly from the field distribution in Figure 4 that the electric fields on upper and lower graphene layers oscillated synchronously. This conclusion can still hold in multilayer graphene-grating structures. Finally, *κ*(*n*, *h*, ∆*θ*) ∝ *e*^*-hq*(*n*)^, where $q\left(n\right)={\left[\beta^{2}\left(n\right)-\varepsilon_{1,2}k_{0}^{2}\right]}^{1/2}$.

Suppose the solution of having the form of *x*_up_ = *x*_down_ = *x*_0_*e*^-*iωt*^ (no phase difference between GSP on neighbor layers), it is found that the resonant frequency became

(13) $$\omega^{2}=\omega_{0}^{2}+\kappa \left(h,n,\Delta \theta \right)-\frac{\gamma^{2}}{2}.$$

When *h* was small (*h* < 4 μm), the larger *κ*(*n*, *h*, ∆*θ*) ∝ *e*^*-h*^ was the larger shift of resonant frequency would be. And obviously, *κ*(*n*, *h*, ∆*θ*) was approaching 0 rapidly when *h* was large enough, which meant that the resonant frequency became a stable value of ${\left(\omega_{0}^{2}-\gamma^{2}/2\right)}^{1/2}$. Otherwise, *κ*(*n*, *h*, ∆*θ*) was also related to the order of GSP. The high order mode had a small skin deep with weak coupling intensity and less blueshift. When *h* tends to be 0, the grating became too thin to excite the surface mode. This was why the absorption disappeared when *h* = 0 in Figure 7.

### Strong absorption in grating-graphene multilayers

**Figure 8:**
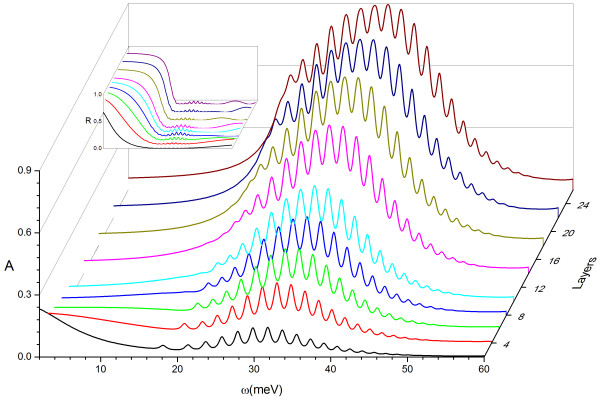
**The absorption spectrums of grating-graphene periodic multilayer structure.** ‘Layers’, number of graphene layers, which is the odd number between 2 and 26. The frequency ranges from 0 to 60 meV (approximately 14.5 THz). The figure inset is the reflections.

Moreover, the behavior of multilayer structures shown in Figure 2b was also investigated using the modified RCWA and the absorption and reflection spectra were given in Figure 8. When increasing the number of graphene layers, it can be seen that the resonant frequencies do not change but for several lower order modes. Though the reflections were always weak within the resonant range, it is obvious that the more graphene layers included, the stronger the absorption is (almost 90% when it contained 26 graphene layers).

**Figure 9:**
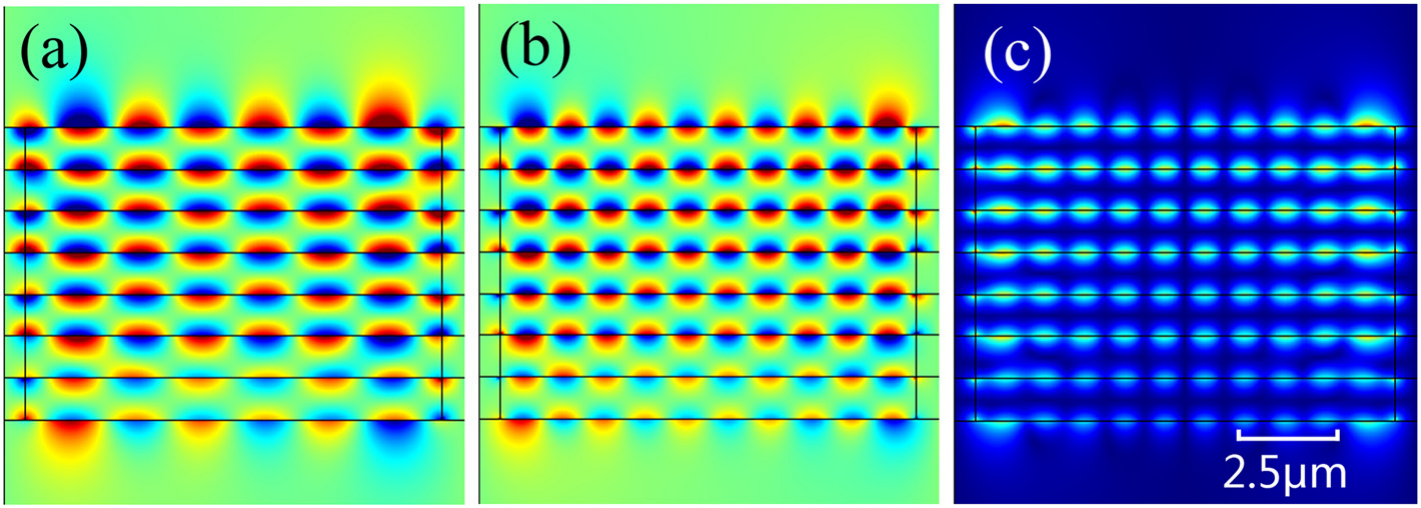
**Field distributions.** The real part **(a)** and **(b)** and magnitude **(c)** of *E*_y_ in multilayer structure of different orders. **(a)** Excitation at the frequency of 24.6 meV. **(b)** and **(c)** Excitation at the frequency of 28.4 meV.

The field distributions of Figure 9 also give the same conclusion that the stand waves on each graphene layer were almost oscillated synchronously. The energy was mainly located and absorbed by the graphene layer as we expected.

## Conclusions

On conclusion, the rigorous coupled wave analysis was modified to compute the excitation of graphene surface plasmon in graphene-containing binary gratings structures. Under the phase matching conditions, the excitation of the graphene surface plasmonics was determined by the distance between graphene layers and duty ratio of gratings, and the mode suppression can be realized by modifying the grating constant and duty ratio. A blueshift of the excitation frequency was obtained for enhanced coupling between GSP of neighbor graphene layers. Increasing the number of graphene layers had almost no effect on the excitation frequency of GSP but would lead to a high absorption with negligible reflection in near-THz range. Finally, the resonant frequency and absorptions can be easily modified by manipulating the structure parameter, including grating constant, duty ratio, and distance between the graphene layers and number of grating, and graphene-containing grating might become potential applications of THz region, such as optical absorption devices, optical nonlinear, optical enhancement, and so on.

## Competing interests

The authors declare that they have no competing interests.

## Authors' contributions

HYU, XJ, and XS conceived the idea. HYU, PL, HYA, and XS wrote the codes, calculated the results, and made the conclusions. HYU, XS, and PL contributed to the preparation and revision of the manuscript. All the authors read and approved the final manuscript.
